# Supplementary material for: A fault-intrusion-tolerant system and deadline-aware algorithm for scheduling scientific workflow in the cloud
Source: PeerJ Comput Sci. 2021 Nov 2;7:e747. doi: 10.7717/peerj-cs.747 (PMC8576554; doi:10.7717/peerj-cs.747)
Supplement: Supplemental Information 6 [file peerj-cs-07-747-s006.zip › Myproject1.1/docs/org/cloudbus/cloudsim/examples/power/planetlab/LrrMmt.html]

LrrMmt


JavaScript is disabled on your browser.


- Overview
- Package
- Class
- Use
- Tree
- Deprecated
- Index
- Help

- Prev Class
- Next Class

- Frames
- No Frames

- All Classes

- Summary:
- Nested |
- Field |
- Constr |
- Method

- Detail:
- Field |
- Constr |
- Method


org.cloudbus.cloudsim.examples.power.planetlab

## Class LrrMmt

- java.lang.Object
- - org.cloudbus.cloudsim.examples.power.planetlab.LrrMmt

- ---

    

  ```
  public class LrrMmt
  extends java.lang.Object
  ```

  A simulation of a heterogeneous power aware data center that applies the Local Regression Robust
  (LRR) VM allocation policy and Minimum Migration Time (MMT) VM selection policy.
  This example uses a real PlanetLab workload: 20110303.
  The remaining configuration parameters are in the Constants and PlanetLabConstants classes.
  If you are using any algorithms, policies or workload included in the power package please cite
  the following paper:
  Anton Beloglazov, and Rajkumar Buyya, "Optimal Online Deterministic Algorithms and Adaptive
  Heuristics for Energy and Performance Efficient Dynamic Consolidation of Virtual Machines in
  Cloud Data Centers", Concurrency and Computation: Practice and Experience (CCPE), Volume 24,
  Issue 13, Pages: 1397-1420, John Wiley & Sons, Ltd, New York, USA, 2012

  Since:
  :   Jan 5, 2012

- - ### Constructor Summary

    Constructors

    Constructor and Description

    `LrrMmt()`
  - ### Method Summary

    Methods

    | Modifier and Type | Method and Description |
    | --- | --- |
    | `static void` | `main(java.lang.String[] args)` The main method. |

    - ### Methods inherited from class java.lang.Object

      `clone, equals, finalize, getClass, hashCode, notify, notifyAll, toString, wait, wait, wait`

- - ### Constructor Detail


    - #### LrrMmt

      ```
      public LrrMmt()
      ```
  - ### Method Detail


    - #### main

      ```
      public static void main(java.lang.String[] args)
                       throws java.io.IOException
      ```

      The main method.

      Parameters:
      :   `args` - the arguments

      Throws:
      :   `java.io.IOException` - Signals that an I/O exception has occurred.


- Overview
- Package
- Class
- Use
- Tree
- Deprecated
- Index
- Help

- Prev Class
- Next Class

- Frames
- No Frames

- All Classes

- Summary:
- Nested |
- Field |
- Constr |
- Method

- Detail:
- Field |
- Constr |
- Method
